# The Impact of Educational Attainment and Income on Long-Term Care for Persons with Alzheimer’s Disease and Other Dementias: A Swedish Nationwide Study

**DOI:** 10.3233/JAD-230388

**Published:** 2023-11-07

**Authors:** Minh Tuan Hoang, Ingemar Kåreholt, Pär Schön, Lena von Koch, Hong Xu, Edwin C.K Tan, Kristina Johnell, Maria Eriksdotter, Sara Garcia-Ptacek

**Affiliations:** a Division of Clinical Geriatrics, Department of Neurobiology, Care Sciences and Society, Karolinska Institutet, Stockholm, Sweden; b Institute of Gerontology, School of Health Welfare, Aging Research Network – Jönköping (ARN-J), Jönköping University, Jönköping, Sweden; c Department of Social Work, Stockholm University, Stockholm, Sweden; d Division of Family Medicine and Primary Care, Department of Neurobiology, Care Sciences and Society, Karolinska Institutet, Stockholm, Sweden; e Neuro Theme, Karolinska University Hospital, Stockholm, Sweden; f The University of Sydney, Faculty of Medicine and Health, School of Pharmacy, New South Wales, Australia; g Department of Medical Epidemiology and Biostatistics, Karolinska Institutet, Stockholm, Sweden; h Aging Theme, Karolinska University Hospital, Stockholm, Sweden

**Keywords:** KeywordsAlzheimer’s disease, aged care, dementia, disparity, education, home care, income, inequality, institutional care, long-term care

## Abstract

**Background:**

Long-term care improves independence and quality of life of persons with dementia (PWD). The influence of socioeconomic status on access to long-term care was understudied.

**Objective:**

To explore the socioeconomic disparity in long-term care for PWD.

**Methods:**

This registry-based study included 14,786 PWD, registered in the Swedish registry for cognitive and dementia disorders (2014–2016). Education and income, two traditional socioeconomic indicators, were the main exposure. Outcomes were any kind of long-term care, specific types of long-term care (home care, institutional care), and the monthly average hours of home care. The association between outcomes and socioeconomic status was examined with zero-inflated negative binomial regression and binary logistic regression.

**Results:**

PWD with compulsory education had lower likelihood of receiving any kind of long-term care (OR 0.80, 95% CI 0.68–0.93), or home care (OR 0.83, 95% CI 0.70–0.97), compared to individuals with university degrees. Their monthly average hours of home care were 0.70 times (95% CI 0.59–0.82) lower than those of persons with university degrees. There was no significant association between education and the receipt of institutional care. Stratifying on persons with Alzheimer’s disease showed significant association between lower education and any kind of long-term care, and between income and the hours of home care.

**Conclusions:**

Socioeconomic inequalities in long-term care existed in this study population. Lower-educated PWD were less likely to acquire general long-term care, home care and had lower hours of home care, compared to their higher-educated counterparts. Income was not significantly associated with the receipt of long-term care.

## INTRODUCTION

Assuring the equal access to long-term care for all persons with dementia is one of the key principles in the “Global action plan on the public health response to dementia” of the World Health Organization [1]. Persons with dementia usually need long-term care because of their disability and dependence in activities of daily living. Long-term care is also important for persons with dementia to improve their quality of life. In Sweden, long-term care for older people is politically governed, predominantly financed, organized, and provided by each of the 290 municipalities. Access to long-term care, which includes home care and institutional care, is based on a needs-assessment (not means-tested) carried out by municipal officers who decide the eligibility, level and range of services [2]. The choice between home care and institutional care largely depends on care needs.

Previous studies showed that access to long-term care was significantly associated with age, gender, race, marital status, type of dementia, the severity of dementia, and living arrangement [3–8]. Ethnicity influenced long-term care placement [4, 9, 10], or was related to delayed dementia care services [11]. Other studies mentioned the time until institutionalization of persons with dementia was predicted by age, gender, race, marital status, severity of cognitive impairment, and mobility impairment [12–18].

In these previous studies, access to home care for persons with dementia was understudied. No studies investigated the amount of home care hours that persons with dementia received. Sweden and other Western European countries aim to decrease the institutionalization for persons with dementia by increasing the provision of home care services [2, 19, 20]. Thus, it is necessary to evaluate long-term care, including both home care and institutional care, for persons with dementia. Meanwhile, the influence of socioeconomic status, which includes education and income, on the receipt of long-term was also less mentioned these preceding studies. Lower socioeconomic status was associated with a higher dementia-related mortality risk [21–24]. Swedish healthcare system with universal health coverage might ameliorate, but probably not eliminate differences in care. Nevertheless, persons with dementia were less likely to receive health care, diagnosis, and treatment if they were from a more disadvantaged socioeconomic status in previous studies [25, 26]. Hence, our study aimed to examine whether socioeconomic status conditioned access to long-term care for persons with dementia, especially persons with Alzheimer’s disease. In this study, we assessed general long-term care, specific home care or institutional care, and the amount of home care hours during the first year after dementia diagnosis.

## MATERIALS AND METHODS

This study was reported according to the REporting of studies Conducted using Observational Routinely collected health Data (RECORD) statement [27] (Supplementary Table 1). The medical ethics were approved by the Swedish Ethical Review Authority (decision number 2017/501-31; 2017/1448-32; 2021-05289).

### Study design and setting

This cross-sectional study used data of persons with dementia registered in the Swedish registry for cognitive and dementia disorders (SveDem) between 2014 and 2016, the Swedish Social Services Register, the Swedish Longitudinal Integrated Database for Health Insurance and Labor Market Studies (LISA), the Swedish National Patient Register, the Swedish Prescribed Drug Register and the Swedish Cause of Death Register. The linkage of these registers was conducted with the Swedish personal identification number by the Swedish National Board of Health and Welfare, and Statistics Sweden. Before delivery for research, the personal identification was pseudonymized and blinded to the researchers.

Established in 2007, SveDem is a nationwide quality of care register, composed of persons with dementia in Sweden who are diagnosed according to the International Classification of Diseases, Tenth Revision [28]. SveDem includes information at baseline registration and annual follow-ups, regarding demographics, cognition, diagnosis, and medication, as previously described [8, 28]. With more than 100,000 patients, SveDem is the largest clinical dementia registry in the world [29].

Data on socioeconomic status one year before dementia diagnosis was extracted from LISA. Founded in 1990, the goal of LISA is to provide a tool for statistical research on health and labor market [30, 31]. This database encompasses information regarding education, employment, and income of all individuals over 15 years old [30, 31].

Data on long-term care was retrieved from the Swedish Social Services Register. This register has been initiated in 2007, including monthly statistics on long-term care for older people and persons with disabilities [32]. Until 2019, about 401,000 older people and 57,200 persons with disabilities who receive at least one form of long-term care have been registered in this registry [33].

Comorbidities, drug prescription and the date of death were collected from Swedish National Patient Register, the Swedish Prescribed Drug Register and the Swedish Cause of Death Register, respectively (Supplementary Table 2).

### Participants

**Fig. 1:**
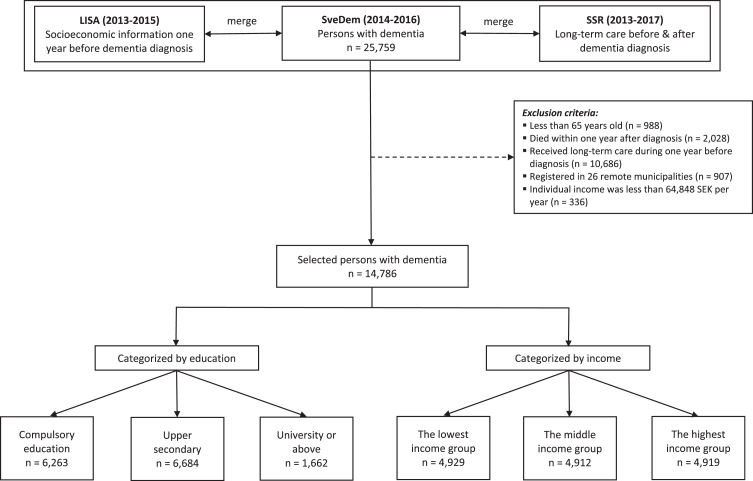
Patient selection. SveDem, the Swedish registry for cognitive and dementia disorders. LISA, the Swedish Longitudinal Integrated Database for Health Insurance and Labor Market Studies. SSR, the Swedish Social Services Register. Education was divided into three categories: compulsory education, upper secondary, and university. Compulsory education in Sweden includes primary school and secondary school (years 1–9). Upper secondary implies high school (years 10–12). University education consists of college, university or higher (master or doctoral education). The lowest income group, annual income was between 64,848 SEK and 161,179 SEK. The middle-income group, annual income was between 161,179 SEK and 204,172 SEK. The highest income group, annual income was more than 204,172 SEK.

The process of selecting participants was depicted in Fig. 1. Data of 25,759 persons with dementia registered in SveDem (2014–2016) were linked with LISA (2013–2015) to retrieve socioeconomic information one year before dementia diagnosis. Inclusion criteria were 1) participants should be 65 years old or older at the time of dementia diagnosis. We assumed that they had less fluctuations in income after this age, since it is the most common retirement age; 2) persons should be still alive one year after dementia diagnosis; 3) since the analyses were on incident care, participants should not receive any kind of long-term care in the year preceding dementia diagnosis. To obtain the data with reliable quality, we only selected people registered in the Swedish Social Services Register between 2013 and 2017. Data between 2007 and 2012 were not employed in our study because the system of data collection was changed, resulting in poor data quality of the Swedish Social Services Register [32]. People registered in 26 (about 9% of total 290) smaller and mostly rural municipalities were also excluded because of unreliable information in these municipalities [32]. Additionally, 336 patients (1.3% of 25,759) who had individual income less than 64,848 SEK per year or negative income were excluded. This threshold is normally the lowest possible income for people from 65 years old, because lower incomes or pensions are complemented up to this level with universal government support for older people [34]. People with lower income than this amount were assumed to live off other assets or savings which would have disqualified them from receiving the minimum financial support for older people. A total of 14,786 persons with dementia was retained for analysis.

### Variables and data sources

Education and individual income, two traditional indicators of socioeconomic status, were the main exposures. Education, extracted from LISA, was the highest educational attainment [30]. Education was divided into three categories: compulsory education, upper secondary, and university [30]. Compulsory education in Sweden includes primary school and secondary school (years 1–9) [30]. Upper secondary implies high school (years 10–12) [30]. University education consists of college, university or higher (master or doctoral education) [30]. Education of immigrants is explored by annual questionnaires [30]. If immigrants participate in any educational activity in Sweden, the new level of education will be recorded and override the older one, via their personal identity number [30]. Individual income, provided by LISA, was defined as the total income that a person received after paying taxes (including all types of income, allowances, or pension) [30]. Income of persons with dementia one year before dementia diagnosis was inflated into 2022 values with inflation rate from the Swedish Consumer Price Index [35]. The inflated income was then divided into three equal groups.

SveDem contributed covariates, including dementia types, types of diagnostic unit, and Mini-Mental State Examination (MMSE) scores at dementia diagnosis. Other socio-demographic covariates included age at dementia diagnosis, sex, living alone, and living areas. Comorbidities before dementia diagnosis, retrieved from the Swedish National Patient Register, were condensed into the Charlson Comorbidity Index [36, 37]. Drug prescription during one year before dementia diagnosis was found from the Swedish Prescribed Drug Register.

Outcomes, extracted from the Swedish Social Services Register, were any kind of long-term care (either institutional care or home care, or both), or a specific type of long-term care (institutional care only or home care only) during one year after dementia diagnosis. Home care was analyzed both as the receipt of care and the monthly average hours of home care.

### Statistical analyses

Categorical variables were presented as number of cases and percentages. Pearson’s Chi-square was employed to compare attributes among different education or income levels. Median, interquartile range (IQR), and *p*-values from Kruskal-Wallis test were used to present numerical variables.

The association between long-term care and socioeconomic status was examined with zero-inflated negative binomial regression (for home care) and binary logistic regression (for any kind of long-term care and institutional care). The zero-inflated negative binomial regression simultaneously implements two separate models: a logit model predicted whether a patient received home care, and a negative binomial model predicted the monthly average hours of home care that persons with dementia received. With each outcome, two models of regression were performed to examine its association with socioeconomic status, as well as the robustness of the results. The first model was controlled for age at dementia diagnosis, sex, living areas, living alone, education or income, Charlson Comorbidity Index, MMSE score, and dementia types. The second model was fully adjusted with the above covariates and additionally controlled for education (if income was the independent variable) or income (if education was the independent variable). Wald test was applied after the regression models to evaluate whether the overall association between outcomes and socioeconomic status was statistically significant or not. Odds ratio (OR), rate ratios (RR), and 95% confidence interval (95% CI) were reported. Sub-group analysis was performed based on age groups, sex, and cohabiting status. We also performed the analysis on persons with Alzheimer’s disease only (*n* = 5,249).

All statistical tests were two tailed with a *p*-value less than 0.05 considered statistically significant. STATA version 17 (copyright StataCorp LLC, College Station, TX, USA) was employed to perform the statistical analyses in this study.

## RESULTS

### Description of the study population

Characteristics of the cohort among different education and income levels were presented in Tables 1 and 2, respectively. The percentage of women decreased with higher education (from 54.0% to 47.4%) and with higher income (from 74.1% to 32.7%). The MMSE scores differed significantly in the groups, increasing in the higher education and income levels. Alzheimer’s disease was the most common dementia type in all education or income levels, with about one-third of the total cohort. More than 50% of persons with dementia in all education or income levels received any kind of long-term care. The proportion of persons with dementia receiving only institutional care was higher in the lower socioeconomic status: declining from 5.9% to 4.1% (corresponding to lowest to highest education levels).

**Table 1:** Characteristics of persons with dementia from different educational levels (*n* = 14,786)

	Compulsory education	Upper secondary	University	*p*
	(*n* = 6,263)	(*n* = 6,684)	(*n* = 1,662)
Age at dementia diagnosis, y, median (IQR)	80.0 (76.0, 84.0)	78.0 (73.0, 83.0)	78.0 (73.0, 83.0)	< 0.001
From 65 to 74	1,298 (20.7)	2109 (31.6)	552 (33.2)	< 0.001
75 and above	4,965 (79.3)	4,575 (68.4)	1,110 (66.8)
Sex, women, *n* (%)	3,382 (54.0)	3,537 (52.9)	787 (47.4)	< 0.001
Municipality types, *n* (%)				< 0.001
Urban	1,563 (25.0)	2,348 (35.2)	807 (48.6)
Intermediate	2,291 (36.6)	2,292 (34.3)	493 (29.7)
Rural	2,403 (38.4)	2,038 (30.5)	359 (21.6)
Living alone, *n* (%)	2,831 (45.2)	2,696 (40.3)	591 (35.6)	< 0.001
Types of dementia diagnostic unit, *n* (%)				< 0.001
Primary care	3,721 (59.4)	2,941 (44.0)	470 (28.3)
Memory clinic	2,542 (40.6)	3,743 (56.0)	1192 (71.7)
MMSE Scores, median (IQR)	21.0 (18.0, 24.0)	22.0 (19.0, 25.0)	24.0 (21.0, 26.0)	< 0.001
Dementia diagnosis, *n* (%)				< 0.001
Alzheimer’s disease	2,014 (32.2)	2,503 (37.5)	689 (41.5)
Mixed dementia	1,096 (17.5)	1,280 (19.2)	324 (19.5)
Vascular dementia	1,196 (19.1)	1,085 (16.2)	240 (14.5)
Lewy body dementia	115 (1.8)	177 (2.7)	53 (3.2)
Frontotemporal dementia	80 (1.3)	120 (1.8)	46 (2.8)
Parkinson disease with dementia	55 (0.9)	102 (1.5)	41 (2.5)
Unspecified dementia	1,541 (24.6)	1,242 (18.6)	231 (13.9)
Other dementias	160 (2.6)	169 (2.5)	35 (2.1)
Charlson Comorbidity Index before dementia diagnosis, median (IQR)	1.0 (0.0, 2.0)	1.0 (0.0, 2.0)	1.0 (0.0, 2.0)	0.15
Comorbidities before dementia diagnosis, *n*(%)
Atrial fibrillation	1,105 (17.6)	1,060 (15.9)	256 (15.4)	0.010
Cancer	1,484 (23.7)	1,701 (25.4)	485 (29.2)	< 0.001
Cerebrovascular diseases	1,181 (18.9)	1,139 (17.0)	285 (17.1)	0.019
Congestive heart failure	642 (10.3)	561 (8.4)	105 (6.3)	< 0.001
Chronic obstructive pulmonary disease	330 (5.3)	314 (4.7)	49 (2.9)	< 0.001
Diabetes	337 (5.4)	296 (4.4)	55 (3.3)	< 0.001
Hypertensive diseases	2,995 (47.8)	2,900 (43.4)	617 (37.1)	< 0.001
Liver diseases	34 (0.5)	51 (0.8)	10 (0.6)	0.29
Myocardial infarction	797 (12.7)	654 (9.8)	133 (8.0)	< 0.001
Peripheral vascular diseases	392 (6.3)	352 (5.3)	55 (3.3)	< 0.001
Renal diseases	215 (3.4)	174 (2.6)	44 (2.6)	0.015
Rheumatic diseases	437 (7.0)	495 (7.4)	108 (6.5)	0.37
Drug prescription during 1 year before dementia diagnosis, *n* (%)
ACEi/ARBs	2,864 (45.7)	2,814 (42.1)	616 (37.1)	< 0.001
Antidepressants	1,662 (26.5)	1,904 (28.5)	505 (30.4)	0.002
Antipsychotics	238 (3.8)	287 (4.3)	59 (3.5)	0.22
Anxiolytics	872 (13.9)	916 (13.7)	196 (11.8)	0.073
Beta blockers	2,491 (39.8)	2,351 (35.2)	512 (30.8)	< 0.001
Calcium channel blockers	1,730 (27.6)	1,637 (24.5)	353 (21.2)	< 0.001
Cholinesterase inhibitors	1,115 (17.8)	1,511 (22.6)	393 (23.6)	< 0.001
Diuretics	1,710 (27.3)	1,432 (21.4)	272 (16.4)	< 0.001
Hypnotics	1,318 (21.0)	1,387 (20.8)	357 (21.5)	0.79
Memantine	168 (2.7)	194 (2.9)	66 (4.0)	0.021
Statins	2,465 (39.4)	2,429 (36.3)	563 (33.9)	< 0.001
Any kind of long-term care, *n* (%)	2,316 (55.4)	2,223 (53.9)	535 (54.1)	0.36
Specific type of long-term care
Institutional care only, *n* (%)	278 (5.9)	208 (4.4)	48 (4.1)	0.001
Home care only, *n* (%)	1,927 (40.3)	1,893 (39.2)	477 (40.9)	0.41
Total hours, median (IQR)	112.0 (39.0, 297.0)	110.0 (40.0, 272.0)	104.0 (38.0, 338.0)	0.98
Monthly average hours, median (IQR)	22.4 (10.0, 44.8)	23.0 (10.3, 46.6)	21.0 (9.0, 45.8)	0.62

Categorical and continuous variables were examined by Chi-square test and Kruskal–Wallis test, respectively. IQR, Inter Quartile Range; MMSE, Mini-Mental State Examination; ACEi/ARBs, Angiotensin-converting enzyme inhibitors / Angiotensin II receptor blockers. Education was divided into three categories: compulsory education, upper secondary, and university. Compulsory education in Sweden includes primary school and secondary school (years 1–9). Upper secondary implies high school (years 10–12). University education consists of college, university or higher (master or doctoral education).

**Table 2:** Characteristics of persons with dementia from different income levels (*n* = 14,786)

	The lowest income group	The middle-income group	The highest income group	*p*
	(*n* = 4,929)	(*n* = 4,912)	(*n* = 4,919)
Age at dementia diagnosis, y, median (IQR)	80.0 (75.0, 84.0)	79.0 (75.0, 84.0)	78.0 (72.0, 83.0)	< 0.001
From 65 to 74	1,160 (23.5)	1,173 (23.9)	1,647 (33.5)	< 0.001
75 and above	3,769 (76.5)	3,739 (76.1)	3,272 (66.5)
Sex, women, *n* (%)	3,650 (74.1)	2,545 (51.8)	1,609 (32.7)	< 0.001
Municipality types, *n* (%)				< 0.001
Urban	1,184 (24.0)	1,438 (29.3)	2,180 (44.4)
Intermediate	1,805 (36.6)	1,793 (36.5)	1,523 (31.0)
Rural	1,937 (39.3)	1,675 (34.1)	1,210 (24.6)
Living alone, *n* (%)	1,673 (33.9)	2,626 (53.5)	1,895 (38.5)	< 0.001
Types of dementia diagnostic unit, *n* (%)				< 0.001
Primary care	2,861 (58.0)	2,622 (53.4)	1,713 (34.8)
Memory clinic	2,068 (42.0)	2,290 (46.6)	3,206 (65.2)
MMSE Scores, median (IQR)	21.0 (18.0, 24.0)	22.0 (18.0, 24.0)	23.0 (20.0, 26.0)	< 0.001
Dementia diagnosis, *n* (%)				< 0.001
Alzheimer’s disease	1,704 (34.6)	1671 (34.1)	1,865 (38.0)
Mixed dementia	829 (16.8)	914 (18.6)	1,003 (20.4)
Vascular dementia	873 (17.7)	887 (18.1)	791 (16.1)
Lewy body dementia	85 (1.7)	97 (2.0)	168 (3.4)
Frontotemporal dementia	64 (1.3)	70 (1.4)	112 (2.3)
Parkinson disease with dementia	35 (0.7)	49 (1.0)	114 (2.3)
Unspecified dementia	1,203 (24.4)	1,098 (22.4)	747 (15.2)
Other dementias	133 (2.7)	120 (2.4)	113 (2.3)
Charlson Comorbidity Index before dementia diagnosis, median (IQR)	1.0 (0.0, 2.0)	1.0 (0.0, 3.0)	1.0 (0.0, 2.0)	0.15
Comorbidities before dementia diagnosis, *n* (%)
Atrial fibrillation	749 (15.2)	808 (16.4)	882 (17.9)	0.001
Cancer	1,055 (21.4)	1,248 (25.4)	1,392 (28.3)	< 0.001
Cerebrovascular diseases	841 (17.1)	896 (18.2)	895 (18.2)	0.22
Congestive heart failure	450 (9.1)	467 (9.5)	402 (8.2)	0.057
Chronic obstructive pulmonary disease	241 (4.9)	264 (5.4)	194 (3.9)	0.003
Diabetes	254 (5.2)	257 (5.2)	189 (3.8)	0.001
Hypertensive diseases	2,278 (46.2)	2,246 (45.7)	2,053 (41.7)	< 0.001
Liver diseases	30 (0.6)	33 (0.7)	33 (0.7)	0.90
Myocardial infarction	493 (10.0)	598 (12.2)	508 (10.3)	< 0.001
Peripheral vascular diseases	234 (4.7)	314 (6.4)	257 (5.2)	0.001
Renal diseases	119 (2.4)	157 (3.2)	161 (3.3)	0.021
Rheumatic diseases	365 (7.4)	363 (7.4)	318 (6.5)	0.11
Drug prescription during 1 year before dementia diagnosis, *n* (%)
ACEi/ARBs	2,169 (44.0)	2,144 (43.6)	2,043 (41.5)	0.028
Antidepressants	1,442 (29.3)	1,359 (27.7)	1,314 (26.7)	0.018
Antipsychotics	213 (4.3)	194 (3.9)	184 (3.7)	0.33
Anxiolytics	762 (15.5)	682 (13.9)	569 (11.6)	< 0.001
Beta blockers	1,894 (38.4)	1,872 (38.1)	1,636 (33.3)	< 0.001
Calcium channel blockers	1,298 (26.3)	1,294 (26.3)	1,168 (23.7)	0.003
Cholinesterase inhibitors	933 (18.9)	997 (20.3)	1,106 (22.5)	< 0.001
Diuretics	1,300 (26.4)	1,242 (25.3)	911 (18.5)	< 0.001
Hypnotics	1,107 (22.5)	1,039 (21.2)	956 (19.4)	0.001
Memantine	137 (2.8)	133 (2.7)	161 (3.3)	0.19
Statins	1,775 (36.0)	1,899 (38.7)	1,839 (37.4)	0.025
Any kind of long-term care, *n* (%)	1,767 (54.4)	1,819 (56.2)	1,547 (53.3)	0.075
Specific type of long-term care
Institutional care only, *n* (%)	197 (5.4)	175 (4.8)	167 (4.9)	0.47
Home care only, *n* (%)	1,458 (39.3)	1,546 (41.4)	1,345 (39.3)	0.091
Total hours, median (IQR)	105.0 (36.0, 281.0)	116.0 (41.0, 298.0)	117.0 (40.0, 311.0)	0.14
Monthly average hours, median (IQR)	22.0 (10.0, 44.8)	24.0 (10.5, 47.6)	22.0 (10.0, 46.8)	0.27

Categorical and continuous variables were examined by Chi-square test and Kruskal–Wallis test, respectively. IQR, Inter Quartile Range; MMSE, Mini-Mental State Examination; ACEi/ARBs, Angiotensin-converting enzyme inhibitors / Angiotensin II receptor blockers. The lowest income group, annual income was between 64,848 SEK and 161,179 SEK. The middle-income group, annual income was between 161,179 SEK and 204,172 SEK. The highest income group, annual income was more than 204,172 SEK.

### The receipt of long-term care in association with education

Table 3 illustrated the association between education and long-term care. Education was significantly associated with the receipt of any kind of long-term care. Persons with dementia with compulsory education had significantly lower likelihood of getting long-term care, compared to persons with dementia with university degrees (OR 0.80, 95% CI 0.68–0.93). The odds of receiving home care were lower in persons with dementia with compulsory education, compared to persons with dementia with university degrees (OR 0.83, 95% CI 0.70–0.97). In comparison with the university degree category, the monthly average hours of home care of the compulsory and upper secondary education categories were 0.70 times lower (95% CI 0.59–0.82) and 0.79 times lower (95% CI 0.68–0.92), respectively.

**Table 3:** Education in association with long-term care for persons with dementia

		Model 1	Model 2
**Any kind of long-term care**	University	reference	reference
	Upper secondary	0.88 (0.76, 1.02)	0.88 (0.76, 1.03)
	Compulsory education	0.80 (0.68, 0.93)^*^	0.80 (0.68, 0.93)^*^
	*p*	0.007	0.010
**Specific type of long-term care**
Institutional care only	University	reference	reference
	Upper secondary	0.84 (0.60, 1.18)	0.88 (0.62, 1.24)
	Compulsory education	0.94 (0.67, 1.32)	1.00 (0.70, 1.43)
	*p*	0.428	0.418
Home care only
*Estimate of use*	University	reference	reference
	Upper secondary	0.88 (0.76, 1.02)	0.89 (0.76, 1.04)
	Compulsory education	0.81 (0.69, 0.95)	0.83 (0.70, 0.97)^*^
	*p*	0.022	0.058
*Monthly average hours*	University	reference	reference
	Upper secondary	0.80 (0.69, 0.92)^*^	0.79 (0.68, 0.92)^*^
	Compulsory education	0.71 (0.61, 0.82)^*^	0.70 (0.59, 0.82)^*^
	*p*	< 0.001	< 0.001

Any kind of long-term care and institutional care were analyzed with binary logistic regression and presented as odds ratio (95% confidence interval). Home care was analyzed with zero-inflated negative binomial regression. The estimate of use was presented as odds ratio (95% confidence interval). The monthly average hours of home care was presented as rate ratio (95% confidence interval). Model 1: Adjusted for age, sex, living areas, living alone, education, Charlson Comorbidity Index, MMSE score, and dementia types. Model 2: Additionally adjusted for disposable individual income. Education was divided into three categories: compulsory education, upper secondary, and university. Compulsory education in Sweden includes primary school and secondary school (years 1–9). Upper secondary implies high school (years 10–12). University education consists of college, university or higher (master or doctoral education). *p*-value was calculated with Wald test to examine the overall significant association of education levels with outcomes. ^*^p-value is less than 0.05.

In the subgroup analyses (Supplementary Tables 3, 5, and 7), the likelihood of receiving any kind of long-term care was significantly lower among lower-educated patients who were between 65 and 74 years old, female or living alone, but not among those aged from 75 years old, male, or living with a partner. Significantly lower number of home care hours in the lower education categories was observed when stratifying by age group, sex, and cohabiting status. Among persons with Alzheimer’s disease, education was significantly associated with the receipt of any kind of long-term care, but not with either institutional care or home care (Supplementary Table 9). Persons with compulsory education was at 0.74 times lower (95% CI 0.56–0.99) of receiving any kind of long-term care, compared to individuals with university degree.

### The receipt of long-term care in association with income

As shown in Table 4, there was no statistically significant association between income and the receipt of any kind of long-term care, institutional care, or home care. The subgroup analysis on age showed lower income was significantly associated with higher chance of receiving institutional care in the patients aged 65–74 years old (OR 1.85, 95% CI 1.02–3.37), or with lower chance of receiving home care in patients aged 75 and above (OR 0.85, 95% CI 0.74–0.97) (Supplementary Table 4). Compared to the highest income group, the monthly average hours of home care in the middle-income group was significantly lower among female patients (RR 0.80, 95% CI 0.69–0.93), but higher among male individuals (RR 1.30, 95% CI 1.09–1.56) (Supplementary Table 6). The monthly average hours of home care of lower-income persons with dementia who lived alone was 1.24 times (95% CI 1.06–1.44) higher, compared to that of higher-income individuals (Supplementary Table 8). Stratifying on persons with Alzheimer’s disease showed significant association between income and the monthly average hours of home care (Supplementary Table 10).

**Table 4:** Income in association with long-term care for persons with dementia

		Model 1	Model 2
**Any kind of long-term care**	The highest income group	reference	reference
	The middle-income group	0.93 (0.83, 1.04)	0.96 (0.86, 1.08)
	The lowest income group	0.97 (0.86, 1.09)	1.02 (0.90, 1.16)
	*p*	0.407	0.564
**Specific type of long-term care**
Institutional care only	The highest income group	reference	reference
	The middle-income group	0.81 (0.64, 1.03)	0.80 (0.63, 1.03)
	The lowest income group	0.92 (0.72, 1.17)	0.91 (0.70, 1.18)
	*p*	0.204	0.202
Home care only
*Estimate of use*	The highest income group	reference	reference
	The middle-income group	0.90 (0.80, 1.01)	0.92 (0.82, 1.04)
	The lowest income group	0.93 (0.82, 1.05)	0.97 (0.85, 1.10)
	*p*	0.185	0.373
*Monthly average hours*	The highest income group	reference	reference
	The middle-income group	0.92 (0.82, 1.02)	1.01 (0.90, 1.13)
	The lowest income group	0.94 (0.84, 1.06)	1.05 (0.93, 1.19)
	*p*-value	0.277	0.691

Any kind of long-term care and institutional care were analyzed with binary logistic regression and presented as odds ratio (95% confidence interval). Home care was analyzed with zero-inflated negative binomial regression. The estimate of use was presented as odds ratio (95% confidence interval). The monthly average hours of home care was presented as rate ratio (95% confidence interval). Model 1: Adjusted for age, sex, living areas, living alone, income, Charlson Comorbidity Index, MMSE score, and dementia types. Model 2: Additionally adjusted for education. The lowest income group, annual income was between 64,848 SEK and 161,179 SEK. The middle-income group, annual income was between 161,179 SEK and 204,172 SEK. The highest income group, annual income was more than 204,172 SEK. *p*-value was calculated with Wald test to examine the overall significant association of education levels with outcomes.

## DISCUSSION

This study aimed to assess the impact of socioeconomic status of persons with dementia on the receipt of long-term care after dementia diagnosis. Persons with dementia with lower education were less likely to receive any kind of long-term care and home care, as well as getting lower home care hours, in comparison to higher-educated persons with dementia. Income was not significantly associated with the receipt of any kind of long-term care or home care. The receipt of institutional care was not significantly associated with either education or income. However, the results diversified when conducting subgroup analysis on age, sex, and cohabiting status.

The socioeconomic inequality in the receipt of home care poses questions to the universal healthcare system. Recently, many countries recommend that persons with dementia live in their own home for as long as possible [20]. There are several reasons for the policy of prioritizing home care instead of institutional care. Persons with dementia living at home had significantly higher quality of life compared to those living in the institutional care [38], and living at home helps persons with dementia maintain their independence [20]. Furthermore, home care is less expensive than institutional care. In Sweden, the cost of institutional care accounted for almost 60% of the societal costs of dementia [39]. Since the 1992 Community Care Reform was implemented, there has been a reduction in length of stay and number of beds in the hospitals, leading to the shift from institutional care to home care in Sweden [2]. A recent study showed that 91,900 out of 158,000 persons with dementia in Sweden (about 58%) lived at home [39]. These facts demonstrate that Sweden has a tradition of supplying long-term care to persons with dementia living at home. Income was not significantly associated with long-term care, suggesting that the low co-pays on a sliding income scale were sufficient to allow everyone access to care, irrespective of income. However, the educational inequalities in the receipt of home care remained. This implies that a universal health- and welfare system, like Sweden, is still not sufficient to ensure the equal access to long-term care for all persons with dementia.

Several factors might contribute to the educational inequalities in the receipt of long-term care and, particularly, home care. The first plausible reason is that higher-educated persons with dementia might be more aware of long-term care, have better communication skills and negotiate better, compared to lower-educated persons with dementia. Additionally, persons with dementia with higher education may have greater expectations for the care provided to them. Thus, they probably seek care earlier and reach the proper care that they need. This explanation is reasonable because our findings showed that higher-educated persons with dementia sought care earlier compared to lower-educated persons with dementia (as can be seen from higher age and lower MMSE at dementia diagnosis of the lower-educated group). Furthermore, normalizing, or stigmatizing views on dementia, which may be more common among lower-educated persons with dementia, might prevent them from reaching long-term care [40, 41]. People with normalizing perspectives consider dementia as a normal and foreseeable part of aging, thus, nothing can prevent dementia [42]. Meanwhile, stigmatizing views presume that dementia is a shameful mental illness that families conceal because of the cultural norm of face-saving [42, 43]. Such perceptions might restrain them from seeking dementia care. An alternative explanation is that living alone possibly influenced the chance of receiving home care. The proportion of persons with dementia living with a partner was higher in the university educated group (Table 1). Persons with dementia living with a partner might have a higher chance of receiving home care because their partners, usually also highly educated, probably help them seek proper care services. Meanwhile, patients living alone might face hurdles to apply for home care services from the municipality.

Our study suggests that persons with dementia living alone should receive more support because they probably have greater difficulties accessing care and no advocates. In our study, persons with dementia with lower education were more frequent in the category of living alone and had worse cognitive function (lower MMSE score), but they were less likely to receive home care, compared to persons with dementia with higher education. This finding is more obvious when we stratified by cohabiting status (Supplementary Table 7). Among persons with dementia living alone, lower education was significantly associated with lower chance of receiving any kind of long-term care (OR 0.68, 95% CI 0.54–0.87) and home care (OR 0.76, 95% CI 0.60–0.97), and getting lower home care hours (RR 0.61, 95% CI 0.50–0.75). Nonetheless, these significant differences were not present among persons with dementia living with a partner. Thus, it might imply that persons with dementia with lower education particularly obtained support from living with other people. In addition, the municipality should take the initiative with persons with dementia, instead of waiting for a request for long-term care from these individuals.

Although we adjusted for living areas in our analysis, socioeconomic disparities in long-term care might also be explained by the geographical location. In this cohort, the proportion of persons with dementia living in the rural areas was higher among the lower-educated category, while the percentage of persons with dementia living in urban areas was higher among the higher-educated group (Table 1). Previous studies showed that older people living in the rural areas had lower chances of being hospitalized or receiving long-term care [44, 45]. Other studies also mentioned that living in more rural areas was significantly associated with unmet needs of dementia care [46, 47]. Population density influences dementia diagnostic work-up [48], and a recent study showed that persons with dementia living in rural areas waited significantly longer time for entering the institutional care [18].

Finally, our findings may denote unequal allocation of health care resources among persons with dementia from different socioeconomic status and unmet needs in persons with dementia with lower socioeconomic status. The Swedish government has implemented policies to reinforce the quality of care for older people and persons with dementia. In 2017, the government promulgated a national plan for quality in health and social care for older people [2]. The main themes in long-term care in this national plan include improving quality and effectiveness, and flexible forms of needs assessments [2]. Ageing in place, which combines primary health care and long-term care, is a policy to target and serve persons with dementia with impaired functioning. Hence, reports and studies on the implementation of these policies should be conducted to evaluate the socioeconomic disparities in long-term care for persons with dementia.

### Limitations

There were several limitations in our study. First, the degree of disability and dependency was not available in this cohort. This is an important confounding factor that might affect the results of the study. The one-year timespan after dementia diagnosis was also a limitation of our study. Previous studies showed that the median time between dementia diagnosis and institutionalization was from 3 to 5 years [14, 16]. Thus, the one-year timespan might underestimate the impact of socioeconomic status on the receipt of institutional care. Additionally, these registers did not include information on informal care from family or partners, underestimating the total care that patients need. In addition, due to the observational study design, causality cannot be inferred, and residual confounding might be present. Finally, even the provision of long-term care by the private sector is minimal in Sweden, the absence of this information is a limitation of this study.

Despite limitations, this study is strengthened by the linkage of national quality registers, with monthly reported long-term care. It enabled the large sample size, ensured the generalizability of the study, and minimized the non-participation bias. Additionally, there was no recall or information bias, which reduced the misclassification of exposure and outcomes. Another advantage is a large sample of persons with dementia from SveDem, which is the largest clinical dementia registry in the world. This is the first study that explores the influence of socioeconomic status on the receipt of long-term care (including both institutional care and home care). This topic is important because home care is increasingly prioritized for older people, especially persons with dementia, in Sweden and other European countries [19, 20].

### Conclusions

To conclude, lower-educated persons with dementia were at lower likelihood of acquiring any kind of long-term care. They also had lower chance of receiving home care, and received significantly lower average hours of home care, compared to their higher-educated counterparts. There were no significant income differences in long-term care. Further studies should be performed to explore reasons for socioeconomic inequalities in long-term care, and to evaluate the experience and expectations with long-term care of persons with dementia or their informal caregivers.

## Supplementary Material

Supplementary Material

## Data Availability

No data are available. The entities responsible for the original data and the Swedish law do not allow for sharing of the data from the Swedish national registers.

## References

[1] World Health Organization, Global action plan on the public health response to dementia 2017 - 2025. https://apps.who.int/iris/bitstream/handle/10665/259615/9789241513487-eng.pdf;jsessionid=F5E1224EBBAC6BD499DA6AE960CFC929?sequence=1, Accessed on January 30, 2023.

[2] Johansson L , Schön P Quality and cost-effectiveness in long-term care and dependency prevention: Country report Sweden. https://aldrecentrum.se/wp-content/uploads/2020/07/quality_and_cost-effectiveness_in_long-term_care_and_d.pdf, Accessed on April 1, 2023.

[3] Banerjee S , Murray J , Foley B , Atkins L , Schneider J , Mann A (2003) Predictors of institutionalisation in people with dementia, J Neurol Neurosurg Psychiatry 74, 1315–1316. 12933944 10.1136/jnnp.74.9.1315 PMC1738636

[4] Cepoiu-Martin M , Tam-Tham H , Patten S , Maxwell CJ , Hogan DB (2016) Predictors of long-term care placement in persons with dementia: A systematic review and meta-analysis, Int J Geriatr Psychiatry 31, 1151–1171. 27045271 10.1002/gps.4449

[5] Cloutier DS , Penning MJ , Nuernberger K , Taylor D , MacDonald S (2019) Long-term care service trajectories and their predictors for persons living with dementia: Results from a Canadian study, J Aging Health 31, 139–164. 28814151 10.1177/0898264317725618

[6] Verbeek H , Meyer G , Challis D , Zabalegui A , Soto ME , Saks K , Leino-Kilpi H , Karlsson S , Hamers JP (2015) Inter-country exploration of factors associated with admission to long-term institutional dementia care: Evidence from the RightTimePlaceCare study, J Adv Nurs 71, 1338–1350. 25869186 10.1111/jan.12663

[7] Gaugler JE , Yu F , Krichbaum K , Wyman JF (2009) Predictors of nursing home admission for persons with dementia, Med Care 47, 191–198. 19169120 10.1097/MLR.0b013e31818457ce

[8] Garcia-Ptacek S , Contreras Escamez B , Zupanic E , Religa D , von Koch L , Johnell K , von Euler M , Kåreholt I , Eriksdotter M (2018) Prestroke mobility and dementia as predictors of stroke outcomes in patients over 65 years of age: A cohort study from the Swedish Dementia and Stroke Registries, J Am Med Direct Assoc 19, 154–161. 10.1016/j.jamda.2017.08.014 28993049

[9] Cooper C , Tandy AR , Balamurali TB , Livingston G (2010) A systematic review and meta-analysis of ethnic differences in use of dementia treatment, care, and research, Am J Geriatr Psychiatry 18, 193–203. 20224516 10.1097/JGP.0b013e3181bf9caf

[10] Stevnsborg L , Jensen-Dahm C , Nielsen TR , Gasse C , Waldemar G (2016) Inequalities in access to treatment and care for patients with dementia and immigrant background: A Danish nationwide study. ,, J Alzheimers Dis 54, 505–514. 27567820 10.3233/JAD-160124

[11] Chin AL , Negash S , Hamilton R (2011) Diversity and disparity in dementia: The impact of ethnoracial differences in Alzheimer disease, Alzheimer Dis Assoc Disord 25, 187–195. 21399486 10.1097/WAD.0b013e318211c6c9 PMC3396146

[12] Gaugler JE , Kane RL , Kane RA , Clay T , Newcomer R (2003) Caregiving and institutionalization of cognitively impaired older people: Utilizing dynamic predictors of change, Gerontologist 43, 219–229. 12677079 10.1093/geront/43.2.219

[13] Luppa M , Riedel-Heller SG , Stein J , Leicht H , König HH , van den Bussche H , Maier W , Scherer M , Bickel H , Mösch E , Werle J , Pentzek M , Fuchs A , Eisele M , Jessen F , Tebarth F , Wiese B , Weyerer S (2012) Predictors of institutionalisation in incident dementia–results of the German Study on Ageing, Cognition and Dementia in Primary Care Patients (AgeCoDe study), Dement Geriatr Cogn Disord 33, 282–288. 22759566 10.1159/000339729

[14] Smith GE , O’Brien PC , Ivnik RJ , Kokmen E , Tangalos EG (2001) Prospective analysis of risk factors for nursing home placement of dementia patients, Neurology 57, 1467–1473. 11673591 10.1212/wnl.57.8.1467

[15] Wattmo C , Wallin AK , Londos E , Minthon L (2011) Risk factors for nursing home placement in Alzheimer’s disease: A longitudinal study of cognition, ADL, service utilization, and cholinesterase inhibitor treatment, Gerontologist 51, 17–27. 20562471 10.1093/geront/gnq050

[16] Luck T , Luppa M , Weber S , Matschinger H , Glaesmer H , Konig HH , Angermeyer MC , Riedel-Heller SG (2008) Time until institutionalization in incident dementia cases–results of the Leipzig Longitudinal Study of the Aged (LEILA 75+), Neuroepidemiology 31, 100–108. 18635941 10.1159/000146251

[17] Korhonen K , Einiö E , Leinonen T , Tarkiainen L , Martikainen P (2018) Time-varying effects of socio-demographic and economic factors on the use of institutional long-term care before dementia-related death: A Finnish register-based study, PLoS One 13, e0199551. 29928067 10.1371/journal.pone.0199551 PMC6013097

[18] Giebel C , Hollinghurst J , Akbari A , Schnier C , Wilkinson T , North L , Gabbay M , Rodgers S (2021) Socio-economic predictors of time to care home admission in people living with dementia in Wales: A routine data linkage study, Int J Geriatr Psychiatry 36, 511–520. 33045103 10.1002/gps.5446 PMC7984448

[19] World Health Organization (2015) World report on ageing and health, World Health Organization, Geneva.

[20] Genet N , Boerma WG , Kringos DS , Bouman A , Francke AL , Fagerström C , Melchiorre MG , Greco C , Devillé W (2011) Home care in Europe: A systematic literature review, BMC Health Serv Res 11, 207–207. 21878111 10.1186/1472-6963-11-207 PMC3170599

[21] van de Vorst IE , Koek HL , Stein CE , Bots ML , Vaartjes I (2016) Socioeconomic disparities and mortality after a diagnosis of dementia: Results from a nationwide registry linkage study, Am J Epidemiol 184, 219–226. 27380760 10.1093/aje/kwv319

[22] Korhonen K , Einio E , Leinonen T , Tarkiainen L , Martikainen P (2020) Midlife socioeconomic position and old-age dementia mortality: A large prospective register-based study from Finland, BMJ Open 10, e033234. 10.1136/bmjopen-2019-033234 PMC6955538 31911519

[23] Strand BH , Langballe EM , Rosness TA , Bergem ALM , Engedal K , Nafstad P , Tell GS , Ormstad H , Tambs K , Bjertness E , group G (2014) Age, education and dementia related deaths. The Norwegian Counties Study and The Cohort of Norway, J Neurol Sci 345, 75–82. 25034053 10.1016/j.jns.2014.07.009

[24] Chen R , Hu Z , Wei L , Wilson K (2014) Socioeconomic status and survival among older adults with dementia and depression, Br J Psychiatry 204, 436–440. 24526747 10.1192/bjp.bp.113.134734

[25] Chen R , Lang L , Clifford A , Chen Y , Hu Z , Han TS (2016) Demographic and socio-economic influences on community-based care and caregivers of people with dementia in China, JRSM Cardiovasc Dis 5, 2048004016652314. 27478589 10.1177/2048004016652314 PMC4948254

[26] Hoang MT , Kåreholt I , von Koch L , Xu H , Secnik J , Religa D , Tan ECK , Johnell K , Garcia-Ptacek S (2021) Influence of education and income on receipt of dementia care in Sweden, J Am Med Dir Assoc 22, 2100–2107. 34280361 10.1016/j.jamda.2021.06.018

[27] Benchimol EI , Smeeth L , Guttmann A , Harron K , Moher D , Petersen I , Sørensen HT , von Elm E , Langan SM (2015) The REporting of studies Conducted using Observational Routinely-collected health Data (RECORD) statement, PLoS Med 12, e1001885. 26440803 10.1371/journal.pmed.1001885 PMC4595218

[28] Religa D , Fereshtehnejad SM , Cermakova P , Edlund AK , Garcia-Ptacek S , Granqvist N , Hallback A , Kawe K , Farahmand B , Kilander L , Mattsson UB , Nagga K , Nordstrom P , Wijk H , Wimo A , Winblad B , Eriksdotter M (2015) SveDem, the Swedish Dementia Registry - a tool for improving the quality of diagnostics, treatment and care of dementia patients in clinical practice, PLoS One 10, e0116538. 25695768 10.1371/journal.pone.0116538 PMC4335024

[29] The Swedish registry for cognitive/dementia disorders - SveDem, SveDem Annual Report 2021, SveDem. https://www.ucr.uu.se/svedem/om-svedem/arsrapporter/arsrapporter/svedem-arsrapport-2021, Accessed on January 30, 2023.

[30] Ludvigsson JF , Svedberg P , Olen O , Bruze G , Neovius M (2019) The longitudinal integrated database for health insurance and labour market studies (LISA) and its use in medical research, Eur J Epidemiol 34, 423–437. 30929112 10.1007/s10654-019-00511-8 PMC6451717

[31] Statistics Sweden, Longitudinal integrated database for health insurance and labour market studies (LISA), Statistics Sweden. http://www.scb.se/en/services/guidance-forresearchers-and-universities/vilka-mikrodata-finns/longitudinella-register/longitudinal-integrated-database-for-health-insurance-and-labour-market-studies-lisa/, Accessed on March 04, 2023.

[32] Meyer AC , Sandström G , Modig K (2022) Nationwide data on home care and care home residence: Presentation of the Swedish Social Service Register, its content and coverage, Scand J Public Health 50, 946–958. 34965796 10.1177/14034948211061016 PMC9578086

[33] The Swedish National Board of Health and Welfare, Statistics on social services for older people. https://www.socialstyrelsen.se/statistik-och-data/statistik/alla-statistikamnen/socialtjanstinsatser-till-aldre/, Accessed on January 30, 2023.

[34] The Swedish Pensions Agency, The elderly care support. https://www.pensionsmyndigheten.se/for-pensionarer/ekonomiskt-stod/ansok-om-aldreforsorjningsstod, Accessed on January 30, 2023.

[35] Statistics Sweden, Consumer Price Index, Statistics Sweden. http://www.scb.se/en/finding-statistics/statisticsby-subject-area/prices-and-consumption/consumer-priceindex/consumer-price-index-cpi/, Accessed on January 30, 2023.

[36] Charlson ME , Pompei P , Ales KL , MacKenzie CR (1987) A new method of classifying prognostic comorbidity in longitudinal studies: Development and validation, J Chronic Dis 40, 373–383. 3558716 10.1016/0021-9681(87)90171-8

[37] Ludvigsson JF , Appelros P , Askling J , Byberg L , Carrero JJ , Ekström AM , Ekström M , Smedby KE , Hagström H , James S , Järvholm B , Michaelsson K , Pedersen NL , Sundelin H , Sundquist K , Sundström J (2021) Adaptation of the Charlson Comorbidity Index for register-based research in Sweden, Clin Epidemiol 13, 21–41. 33469380 10.2147/CLEP.S282475 PMC7812935

[38] Olsen C , Pedersen I , Bergland A , Enders-Slegers M-J , Jøranson N , Calogiuri G , Ihlebæk C (2016) Differences in quality of life in home-dwelling persons and nursing home residents with dementia - a cross-sectional study, BMC Geriatr 16, 137–137. 27400744 10.1186/s12877-016-0312-4 PMC4939817

[39] Wimo A , Jönsson L , Fratiglioni L , Sandman PO , Gustavsson A , Sköldunger A , Johansson L (2016) The societal costs of dementia in Sweden 2012 –relevance and methodological challenges in valuing informal care, Alzheimers Res Ther 8, 59. 27986093 10.1186/s13195-016-0215-9 PMC5162098

[40] Nielsen TR , Waldemar G (2016) Knowledge and perceptions of dementia and Alzheimer’s disease in four ethnic groups in Copenhagen, Denmark , Int J Geriatr Psychiatry 31, 222–230. 26040575 10.1002/gps.4314

[41] Sagbakken M , Spilker RS , Nielsen TR (2018) Dementia and immigrant groups: A qualitative study of challenges related to identifying, assessing, and diagnosing dementia, BMC Health Serv Res 18, 910. 30497459 10.1186/s12913-018-3720-7 PMC6267848

[42] Kovaleva M , Jones A , Maxwell CA (2021) Immigrants and dementia: Literature update, Geriatr Nurs 42, 1218–1221. 34090727 10.1016/j.gerinurse.2021.04.019

[43] Kong EH , Deatrick JA , Evans LK (2010) The experiences of Korean immigrant caregivers of non-English-speaking older relatives with dementia in American nursing homes, Qual Health Res 20, 319–329. 19940089 10.1177/1049732309354279

[44] Virnig BA , Kind S , McBean M , Fisher E (2000) Geographic variation in hospice use prior to death, J Am Geriatr Soc 48, 1117–1125. 10983913 10.1111/j.1532-5415.2000.tb04789.x

[45] Roheger M , Zupanic E , Kåreholt I , Religa D , Kalbe E , Eriksdotter M , Garcia-Ptacek S (2018) Mortality and nursing home placement of dementia patients in rural and urban areas: A cohort study from the Swedish Dementia Registry, Scand J Caring Sci 32, 1308–1313. 29656469 10.1111/scs.12574

[46] Bauer M , Fetherstonhaugh D , Blackberry I , Farmer J , Wilding C (2019) Identifying support needs to improve rural dementia services for people with dementia and their carers: A consultation study in Victoria, Australia, Aust J Rural Health 27, 22–27. 30719789 10.1111/ajr.12444

[47] Morgan D , Innes A , Kosteniuk J (2011) Dementia care in rural and remote settings: A systematic review of formal or paid care, Maturitas 68, 17–33. 21041045 10.1016/j.maturitas.2010.09.008

[48] Roheger M , Eriksdotter M , Westling K , Kalbe E , Garcia-Ptacek S (2019) Basic diagnostic work-up is more complete in rural than in urban areas for patients with dementia: Results of a Swedish Dementia Registry Study, J Alzheimers Dis 69, 455–462. 30988239 10.3233/JAD-190017 PMC6597969

