# Supplementary material for: The Impact of Educational Attainment and Income on Long-Term Care for Persons with Alzheimer’s Disease and Other Dementias: A Swedish Nationwide Study
Source: J Alzheimers Dis. 2023 Nov 7;96(2):789–800. doi: 10.3233/JAD-230388 (PMC10657673; doi:10.3233/JAD-230388)
Supplement: Supplementary Material [file jad-96-jad230388-s001.pdf]

Supplementary Material

The Impact of Educational Attainment and Income on Long-Term Care for Persons with Alzheimer's Disease and Other Dementias: A Swedish Nationwide Study

Supplementary Table 1. The RECORD statement – checklist of items, extended from the STROBE statement, that should be reported in observational studies using routinely collected health data [1]

RECORD items		Details	Location
**Title and abstract**	1	(1) The type of data used should be specified in the title or abstract. When possible, the name of the databases used should be included.	Abstract
		(2) If applicable, the geographic region and timeframe within which the study took place should be reported in the title or abstract.	Abstract
		(3) If linkage between databases was conducted for the study, this should be clearly stated in the title or abstract.	Abstract
**Introduction**			
Background/rationale	2	Explain the scientific background and rationale for the investigation being reported	Introduction > 1st and 2nd paragraphs
Objectives	3	State specific objectives, including any prespecified hypotheses	Introduction > Last paragraph
**Methods**			
Study design	4	Present key elements of study design early in the paper	Methods > Study design and setting
Setting	5	Describe the setting, locations, and relevant dates, including periods of recruitment, exposure, follow-up, and data collection	Methods > Study design and setting
Participants	6	(*1*) The methods of study population selection (such as codes or algorithms used to identify subjects) should be listed in detail. If this is not possible, an explanation should be provided.	Methods > Participants
		(*2*) Any validation studies of the codes or algorithms used to select the population should be referenced. If validation was conducted for this study and not published elsewhere, detailed methods and results should be provided.	Not applicable
		(3) If the study involved linkage of databases, consider use of a flow diagram or other graphical display to demonstrate the data linkage process, including the number of individuals with linked data at each stage.	Figure 1
Variables	7	A complete list of codes and algorithms used to classify exposures, outcomes, confounders, and effect modifiers should be provided. If these cannot be reported, an explanation should be provided.	Methods > Variables & Data sources
Data sources/ measurement	8*	For each variable of interest, give sources of data and details of methods of assessment (measurement). Describe comparability of assessment methods if there is more than one group	Methods > Variables & Data sources
Bias	9	Describe any efforts to address potential sources of bias	Discussion > Limitations
Study size	10	Explain how the study size was arrived at	Methods > Participants & Figure 1
Quantitative variables	11	Explain how quantitative variables were handled in the analyses. If applicable, describe which groupings were chosen and why	Methods > Variables & Data sources
Statistical methods	12	(*a*) Describe all statistical methods, including those used to control for confounding	Methods > Statistical analysis

		(*b*) Describe any methods used to examine subgroups and interactions	Methods > Statistical analysis
		(*c*) Explain how missing data were addressed	Not applicable
		(*d*) If applicable, explain how loss to follow-up was addressed	Not applicable
		(*e*) Describe any sensitivity analyses	Methods > Statistical analysis
Data access and cleaning methods		Authors should describe the extent to which the investigators had access to the database population used to create the study population.	Methods > Study design and data sources & Supplementary Table 2
		Authors should provide information on the data cleaning methods used in the study.	Not applicable
		State whether the study included person-level, institutional-level, or other data linkage across two or more databases. The methods of linkage and methods of linkage quality evaluation should be provided.	Methods > Study design and setting
**Results**			
Participants	13*	(a) Describe in detail the selection of the persons included in the study (i.e., study population selection) including filtering based on data quality, data availability and linkage. The selection of included persons can be described in the text and/or by means of the study flow diagram.	Methods > Participants & Figure 1
		(b) Give reasons for non-participation at each stage	Methods > Participants & Figure 1
		(c) Consider use of a flow diagram	Methods > Participants & Figure 1
Descriptive data	14*	(a) Give characteristics of study participants (e.g., demographic, clinical, social) and information on exposures and potential confounders	Results > Description of the Study Population & Tables 1-2
		(b) Indicate number of participants with missing data for each variable of interest	Not applicable
		(c) Summarise follow-up time (e.g., average and total amount)	Methods > Participants & Figure 1
Outcome data	15*	Report numbers of outcome events or summary measures over time	Results > Tables 1-2
Main results	16	(*a*) Give unadjusted estimates and, if applicable, confounder-adjusted estimates and their precision (e.g., 95% confidence interval). Make clear which confounders were adjusted for and why they were included	Results > Tables 3-4
		(*b*) Report category boundaries when continuous variables were categorized	Not applicable
		(*c*) If relevant, consider translating estimates of relative risk into absolute risk for a meaningful time period	Not applicable
Other analyses	17	Report other analyses done—e.g., analyses of subgroups and interactions, and sensitivity analyses	Supplementary Tables 3-8
**Discussion**			
Key results	18	Summarise key results with reference to study objectives	Results & Discussion > First paragraph
Limitations	19	Discuss the implications of using data that were not created or collected to answer the specific research question(s). Include discussion of misclassification bias, unmeasured confounding, missing data, and changing eligibility over time, as they pertain to the study being reported.	Discussion > Limitations
Interpretation	20	Give a cautious overall interpretation of results considering objectives, limitations, multiplicity of analyses, results from similar studies, and other relevant evidence	Discussion
Generalisability	21	Discuss the generalisability (external validity) of the study results	Discussion
**Other information**			
Funding	22	Give the source of funding and the role of the funders for the present study and, if applicable, for the original study on which the present article is based	Funding sources

Accessibility of protocol, raw data, and programming code	Authors should provide information on how to access any supplemental information such as the study protocol, raw data, or programming code.	Data availability statement

Supplementary Table 2. ICD-10 Codes for comorbidities & ATC Codes for drugs

Comorbidities	ICD-10 Codes [2]
Atrial fibrillation	I48
Cancer	C00 – C97
Cerebrovascular diseases	G45,<br>I60, I61, I62, I63, I64, I67, I69
Congestive heart failure	I110, I130, I132, I255, I420, I426, I427, I428, I429, I43, I50
Chronic obstructive pulmonary disease	J43, J44
Diabetes	E100 – E107, E110 – E117, E120 – E127, E130 -E137, E140 – E147
Hypertensive diseases	I10, I11, I12, I13, I14, I15, I16
Liver diseases	B15, B16, B17, B18, B19,<br>K754, K746, K73, K703, K709,<br>I850, I859, I982, I983
Myocardial infarction	I21, I22, I252
Peripheral vascular diseases	I70, I71, I731, I738, I739, I771, I790, I792,<br>K55
Renal diseases	I120, I131,<br>Z992, Z940, Z49,<br>Q614, Q613, Q612, Q611,<br>N250, N19, N18, N11, N057, N056, N055, N054, N053, N052, N037, N036, N035, N034, N033, N032, Z992
Rheumatic diseases	M05, M06, M070, M071, M072, M073, M08, M123, M13, M30, M313, M314, M315, M316, M32, M33, M34, M350, M351, M353, M45, M46

Drugs	ATC codes [3]
ACEi/ARBs	C09
Antidepressants	N06A
Antipsychotics	N05A
Anxiolytics	N05B
Beta blockers	C07
Calcium channel blockers	C08
Cholinesterase inhibitors	N06DA
Diuretics	C03
Hypnotics	N05C
Memantine	N06DX01
Statins	C10AA

ICD, International Statistical Classification of Diseases and Related Health Problems. 10th revision; ATC, Anatomical Therapeutic Chemical Classification System

Supplementary Table 3. Education in association with long-term care for persons with dementia, stratified by age at dementia diagnosis

		**Aged 65-74 (n = 3,989)**		**Aged 75 or above (n = 10,797)**	
		**Model 1**	**Model 2**	**Model 1**	**Model 2**
**Any kind of long-term care**	University	reference	reference	reference	reference
	Upper secondary	0.85 (0.64, 1.14)	0.85 (0.63, 1.14)	0.89 (0.75, 1.05)	0.89 (0.74, 1.06)
	Compulsory education	0.71 (0.52, 0.97) *	0.71 (0.51, 0.98) *	0.85 (0.71, 1.01)	0.84 (0.70, 1.01)
	p	0.075	0.082	0.167	0.177
**Specific type of long-term care**					
Institutional care only	University	reference	reference	reference	reference
	Upper secondary	0.97 (0.48, 2.00)	0.88 (0.42, 1.86)	0.81 (0.55, 1.19)	0.87 (0.59, 1.29)
	Compulsory education	0.95 (0.44, 2.05)	0.82 (0.37, 1.84)	0.98 (0.67, 1.43)	1.10 (0.74, 1.63)
	p	0.992	0.886	0.200	0.133
Home care only					
Estimate of use	University	reference	reference	reference	reference
	Upper secondary	0.86 (0.65, 1.15)	0.84 (0.63, 1.14)	0.86 (0.65, 1.15)	0.84 (0.63, 1.14)
	Compulsory education	0.76 (0.55, 1.04)	0.74 (0.53, 1.03)	0.76 (0.55, 1.04)	0.74 (0.53, 1.03)
	p	0.210	0.180	0.247	0.481
Monthly average hours	University				
	Upper secondary	0.66 (0.49, 0.89) *	0.66 (0.48, 0.89) *	0.66 (0.49, 0.89) *	0.66 (0.48, 0.89) *
	Compulsory education	0.55 (0.40, 0.75) *	0.53 (0.38, 0.74) *	0.55 (0.40, 0.75) *	0.53 (0.38, 0.74) *
	p-value	0.001	0.001	0.003	0.003

Any kind of long-term care and institutional care were analyzed with binary logistic regression and presented as odds ratio (95% confidence interval).
Home care was analyzed with zero-inflated negative binomial regression. The estimate of use was presented as odds ratio (95% confidence interval). The monthly average hours of home care was presented as rate ratio (95% confidence interval).
Model 1: Adjusted for sex, living areas, living alone, education, Charlson Comorbidity Index, MMSE score, and dementia types.
Model 2: Additionally adjusted for disposable individual income.
Education was divided into three categories: compulsory education, upper secondary, and university. Compulsory education in Sweden includes primary school and secondary school (years 1-9). Upper secondary implies high school (years 10-12). University education consists of college, university or higher (master or doctoral education).
p-value was calculated with Wald test to examine the overall significant association of education levels with outcomes.
* p-value is less than 0.05

Supplementary Table 4. Income in association with long-term care for persons with dementia, stratified by age at dementia diagnosis

		Aged 65-74 (n = 3,989)		Aged 75 or above (n = 10,797)	
		**Model 1**	**Model 2**	**Model 1**	**Model 2**
**Any kind of long-term care**	The highest income group	reference	reference	reference	reference
	The middle-income group	1.07 (0.85, 1.35)	1.13 (0.89, 1.44)	0.90 (0.79, 1.02)	0.92 (0.81, 1.05)
	The lowest income group	0.88 (0.69, 1.13)	0.96 (0.74, 1.24)	1.02 (0.89, 1.17)	1.05 (0.91, 1.21)
	p	0.289	0.356	0.092	0.108
**Specific type of long-term care**					
Institutional care only	The highest income group	reference	reference	reference	reference
	The middle-income group	0.77 (0.42, 1.43)	0.80 (0.43, 1.51)	0.81 (0.62, 1.04)	0.78 (0.60, 1.02)
	The lowest income group	1.77 (1.01, 3.12) *	1.85 (1.02, 3.37) *	0.80 (0.61, 1.06)	0.77 (0.58, 1.03)
	p	0.016	0.014	0.188	0.135
Home care only					
Estimate of use	The highest income group	reference	reference	reference	reference
	The middle-income group	1.12 (0.88, 1.42)	1.17 (0.92, 1.50)	0.84 (0.74, 0.96) *	0.85 (0.74, 0.97) *
	The lowest income group	0.96 (0.74, 1.24)	1.02 (0.78, 1.34)	0.94 (0.81, 1.08)	0.95 (0.82, 1.10)
	p	0.425	0.382	0.031	0.047
Monthly average hours	The highest income group				
	The middle-income group	1.06 (0.83, 1.36)	1.22 (0.94, 1.57)	0.90 (0.80, 1.02)	0.99 (0.87, 1.12)
	The lowest income group	0.82 (0.63, 1.07)	0.95 (0.72, 1.25)	0.99 (0.87, 1.13)	1.10 (0.95, 1.26)
	p	0.146	0.133	0.168	0.215

Any kind of long-term care and institutional care were analyzed with binary logistic regression and presented as odds ratio (95% confidence interval).
Home care was analyzed with zero-inflated negative binomial regression. The estimate of use was presented as odds ratio (95% confidence interval). The monthly average hours of home care was presented as rate ratio (95% confidence interval).
Model 1: Adjusted for sex, living areas, living alone, income, Charlson Comorbidity Index, MMSE score, and dementia types.
Model 2: Additionally adjusted for education
The lowest income group, annual income was between 64,848 SEK and 161,179 SEK. The middle-income group, annual income was between 161,179 SEK and 204,172 SEK. The highest income group, annual income was more than 204,172 SEK.
p-value was calculated with Wald test to examine the overall significant association of education levels with outcomes.
* p-value is less than 0.05

Supplementary Table 5. Education in association with long-term care for persons with dementia, stratified by sex

		Women (n = 7,819)		Men (n = 6,967)	
		**Model 1**	**Model 2**	**Model 1**	**Model 2**
**Any kind of long-term care**	University	reference	reference	reference	reference
	Upper secondary	0.86 (0.70, 1.05)	0.85 (0.69, 1.05)	0.90 (0.72, 1.11)	0.91 (0.73, 1.14)
	Compulsory education	0.76 (0.62, 0.94) *	0.75 (0.60, 0.94) *	0.83 (0.66, 1.04)	0.86 (0.68, 1.08)
	p	0.023	0.024	0.240	0.402
**Specific type of long-term care**					
Institutional care only	University	reference	reference	reference	reference
	Upper secondary	0.65 (0.41, 1.01)	0.69 (0.44, 1.09)	1.13 (0.67, 1.92)	1.17 (0.69, 2.00)
	Compulsory education	0.75 (0.48, 1.17)	0.82 (0.51, 1.31)	1.21 (0.71, 2.05)	1.27 (0.74, 2.20)
	p	0.137	0.203	0.754	0.667
Home care only					
Estimate of use	University	reference	reference	reference	reference
	Upper secondary	0.95 (0.77, 1.17)	0.96 (0.77, 1.19)	0.80 (0.64, 0.99)	0.83 (0.66, 1.04)
	Compulsory education	0.83 (0.67, 1.03)	0.84 (0.67, 1.05)	0.80 (0.63, 1.01)	0.84 (0.66, 1.07)
	p	0.075	0.091	0.132	0.268
Monthly average hours	University				
	Upper secondary	0.86 (0.71, 1.02)	0.90 (0.75, 1.09)	0.71 (0.56, 0.91) *	0.66 (0.51, 0.84) *
	Compulsory education	0.73 (0.61, 0.88) *	0.79 (0.64, 0.96) *	0.67 (0.52, 0.87) *	0.60 (0.46, 0.79) *
	p	0.001	0.018	0.007	<0.001

Any kind of long-term care and institutional care were analyzed with binary logistic regression and presented as odds ratio (95% confidence interval).
Home care was analyzed with zero-inflated negative binomial regression. The estimate of use was presented as odds ratio (95% confidence interval). The monthly average hours of home care was presented as rate ratio (95% confidence interval).
Model 1: Adjusted for age, living areas, living alone, education, Charlson Comorbidity Index, MMSE score, and dementia types.
Model 2: Additionally adjusted for disposable individual income.
Education was divided into three categories: compulsory education, upper secondary, and university. Compulsory education in Sweden includes primary school and secondary school (years 1-9). Upper secondary implies high school (years 10-12). University education consists of college, university or higher (master or doctoral education).
p-value was calculated with Wald test to examine the overall significant association of education levels with outcomes.
* p-value is less than 0.05

Supplementary Table 6. Income in association with long-term care for persons with dementia, stratified by sex

		Women (n = 7,819)		Men (n = 6,967)	
		Model 1	Model 2	Model 1	Model 2
**Any kind of long-term care**	The highest income group	reference	reference	reference	reference
	The middle-income group	0.99 (0.84, 1.17)	1.04 (0.88, 1.22)	0.87 (0.75, 1.02)	0.90 (0.77, 1.05)
	The lowest income group	0.97 (0.82, 1.14)	1.04 (0.87, 1.23)	0.98 (0.81, 1.17)	1.01 (0.83, 1.22)
	p	0.906	0.908	0.203	0.327
**Specific type of long-term care**					
Institutional care only	The highest income group	reference	reference	reference	reference
	The middle-income group	0.72 (0.51, 1.02)	0.73 (0.51, 1.05)	0.89 (0.64, 1.22)	0.86 (0.61, 1.19)
	The lowest income group	0.78 (0.55, 1.11)	0.81 (0.56, 1.17)	0.99 (0.69, 1.44)	0.98 (0.67, 1.44)
	p	0.176	0.241	0.728	0.607
Home care only					
Estimate of use	The highest income group	reference	reference	reference	reference
	The middle-income group	0.94 (0.80, 1.11)	0.96 (0.80, 1.13)	0.86 (0.73, 1.01)	0.88 (0.75, 1.04)
	The lowest income group	0.94 (0.79, 1.11)	0.97 (0.81, 1.16)	0.92 (0.76, 1.13)	0.94 (0.77, 1.16)
	p	0.720	0.870	0.198	0.347
Monthly average hours	The highest income group				
	The middle-income group	0.74 (0.64, 0.85) *	0.80 (0.69, 0.93) *	1.16 (0.97, 1.38)	1.30 (1.09, 1.56) *
	The lowest income group	0.85 (0.73, 0.98) *	0.93 (0.79, 1.09)	0.95 (0.76, 1.17)	1.06 (0.85, 1.33)
	p	<0.001	<0.001	0.128	0.014

Any kind of long-term care and institutional care were analyzed with binary logistic regression and presented as odds ratio (95% confidence interval).
Home care was analyzed with zero-inflated negative binomial regression. The estimate of use was presented as odds ratio (95% confidence interval). The monthly average hours of home care was presented as rate ratio (95% confidence interval).
Model 1: Adjusted for age, living areas, living alone, income, Charlson Comorbidity Index, MMSE score, and dementia types.
Model 2: Additionally adjusted for education
The lowest income group, annual income was between 64,848 SEK and 161,179 SEK. The middle-income group, annual income was between between 161,179 SEK and 204,172 SEK. The highest income group, annual income was more than 204,172 SEK.
p-value was calculated with Wald test to examine the overall significant association of education levels with outcomes.
* p-value is less than 0.05

Supplementary Table 7. Education in association with long-term care for persons with dementia, stratified by living alone versus cohabiting

		Cohabiting (n = 8,566)		Living alone (n = 6,194)	
		**Model 1**	**Model 2**	**Model 1**	**Model 2**
**Any kind of long-term care**	University	reference	reference	reference	reference
	Upper secondary	1.03 (0.84, 1.27)	1.04 (0.84, 1.28)	0.72 (0.57, 0.90) *	0.72 (0.57, 0.90) *
	Compulsory education	0.89 (0.72, 1.10)	0.89 (0.71, 1.11)	0.68 (0.54, 0.85) *	0.68 (0.54, 0.87) *
	p	0.083	0.094	0.004	0.007
**Specific type of long-term care**					
Institutional care only	University	reference	reference	reference	reference
	Upper secondary	0.97 (0.58, 1.60)	1.03 (0.62, 1.73)	0.72 (0.45, 1.14)	0.74 (0.46, 1.18)
	Compulsory education	1.04 (0.63, 1.73)	1.14 (0.67, 1.96)	0.84 (0.53, 1.33)	0.87 (0.54, 1.40)
	p	0.894	0.783	0.277	0.287
Home care only					
Estimate of use	University	reference	reference	reference	reference
	Upper secondary	0.98 (0.79, 1.21)	0.98 (0.79, 1.22)	0.76 (0.61, 0.95) *	0.79 (0.62, 0.99) *
	Compulsory education	0.88 (0.71, 1.10)	0.88 (0.69, 1.11)	0.72 (0.57, 0.91) *	0.76 (0.60, 0.97) *
	p	0.273	0.281	0.025	0.082
Monthly average hours	University				
	Upper secondary	0.93 (0.73, 1.18)	0.95 (0.73, 1.23)	0.70 (0.59, 0.84) *	0.69 (0.57, 0.83) *
	Compulsory education	0.76 (0.59, 0.98) *	0.80 (0.61, 1.06)	0.64 (0.53, 0.77) *	0.61 (0.50, 0.75) *
	p	0.025	0.096	<0.001	<0.001

Any kind of long-term care and institutional care were analyzed with binary logistic regression and presented as odds ratio (95% confidence interval).
Home care was analyzed with zero-inflated negative binomial regression. The estimate of use was presented as odds ratio (95% confidence interval). The monthly average hours of home care was presented as rate ratio (95% confidence interval).
Model 1: Adjusted for age, sex, living areas, education, Charlson Comorbidity Index, MMSE score, and dementia types.
Model 2: Additionally adjusted for disposable individual income.
Education was divided into three categories: compulsory education, upper secondary, and university. Compulsory education in Sweden includes primary school and secondary school (years 1-9). Upper secondary implies high school (years 10-12). University education consists of college, university or higher (master or doctoral education).
p-value was calculated with Wald test to examine the overall significant association of education levels with outcomes.
* p-value is less than 0.05

Supplementary Table 8. Income in association with long-term care for persons with dementia, stratified by living alone versus cohabiting

		Cohabiting (n = 8,566)		Living alone (n = 6,194)	
		**Model 1**	**Model 2**	**Model 1**	**Model 2**
**Any kind of long-term care**	The highest income group	reference	reference	reference	reference
	The middle-income group	0.93 (0.79, 1.10)	0.95 (0.80, 1.13)	0.93 (0.80, 1.08)	0.98 (0.84, 1.14)
	The lowest income group	0.98 (0.82, 1.16)	1.01 (0.84, 1.21)	0.93 (0.78, 1.11)	0.99 (0.83, 1.19)
	p	0.717	0.769	0.585	0.965
**Specific type of long-term care**					
Institutional care only	The highest income group	reference	reference	reference	reference
	The middle-income group	0.66 (0.44, 0.98) *	0.65 (0.43, 0.97) *	0.92 (0.68, 1.24)	0.92 (0.67, 1.26)
	The lowest income group	0.89 (0.61, 1.30)	0.86 (0.57, 1.29)	0.90 (0.64, 1.26)	0.91 (0.64, 1.30)
	p	0.107	0.104	0.805	0.851
Home care only					
Estimate of use	The highest income group	reference	reference	reference	reference
	The middle-income group	0.92 (0.77, 1.09)	0.93 (0.78, 1.12)	0.88 (0.75, 1.02)	0.91 (0.77, 1.06)
	The lowest income group	1.01 (0.85, 1.22)	1.04 (0.86, 1.26)	0.82 (0.69, 0.98) *	0.86 (0.72, 1.03)
	p	0.480	0.472	0.082	0.252
Monthly average hours	The highest income group				
	The middle-income group	1.03 (0.84, 1.26)	1.09 (0.88, 1.36)	0.88 (0.78, 1.01)	1.01 (0.88, 1.15)
	The lowest income group	0.75 (0.60, 0.93) *	0.81 (0.64, 1.03)	1.08 (0.93, 1.25)	1.24 (1.06, 1.44) *
	p	0.008	0.029	0.007	0.004

Any kind of long-term care and institutional care were analyzed with binary logistic regression and presented as odds ratio (95% confidence interval).
Home care was analyzed with zero-inflated negative binomial regression. The estimate of use was presented as odds ratio (95% confidence interval). The monthly average hours of home care was presented as rate ratio (95% confidence interval).
Model 1: Adjusted for age, sex, living areas, income, Charlson Comorbidity Index, MMSE score, and dementia types.
Model 2: Additionally adjusted for education
The lowest income group, annual income was between 64,848 SEK and 161,179 SEK. The middle-income group, annual income was between between 161,179 SEK and 204,172 SEK. The highest income group, annual income was more than 204,172 SEK.
p-value was calculated with Wald test to examine the overall significant association of education levels with outcomes.
* p-value is less than 0.05

Supplementary Table 9. Education in association with long-term care for persons with Alzheimer's disease

		Model 1	Model 2
**Any kind of long-term care**	University	reference	reference
	Upper secondary	0.95 (0.73, 1.22)	0.93 (0.71, 1.20)
	Compulsory education	0.77 (0.59, 1.01)	0.74 (0.56, 0.99) *
	p	0.037	0.027
**Specific type of long-term care**			
Institutional care only	University	reference	reference
	Upper secondary	0.83 (0.46, 1.51)	0.89 (0.48, 1.64)
	Compulsory education	0.83 (0.45, 1.51)	0.91 (0.48, 1.72)
	p	0.813	0.930
Home care only			
Estimate of use	University	reference	reference
	Upper secondary	0.86 (0.66, 1.13)	0.84 (0.64, 1.10)
	Compulsory education	0.75 (0.57, 0.99) *	0.72 (0.54, 0.96) *
	p	0.091	0.066
Monthly average hours	University		
	Upper secondary	0.95 (0.74, 1.22)	1.05 (0.80, 1.37)
	Compulsory education	0.86 (0.66, 1.12)	0.92 (0.70, 1.22)
	p	0.381	0.333

Any kind of long-term care and institutional care were analyzed with binary logistic regression and presented as odds ratio (95% confidence interval). Home care was analyzed with zero-inflated negative binomial regression. The estimate of use was presented as odds ratio (95% confidence interval). The monthly average hours of home care was presented as rate ratio (95% confidence interval).
Model 1: Adjusted for age, sex, living areas, living alone, education, Charlson Comorbidity Index, MMSE score.
Model 2: Additionally adjusted for disposable individual income.
Education was divided into three categories: compulsory education (n = 2014), upper secondary (n = 2503), and university (n = 689). Compulsory education in Sweden includes primary school and secondary school (years 1-9). Upper secondary implies high school (years 10-12). University education consists of college, university or higher (master or doctoral education).
p-value was calculated with Wald test to examine the overall significant association of education levels with outcomes.
* p-value is less than 0.05

Supplementary Table 10. Income in association with long-term care for persons with Alzheimer's disease

		Model 1	Model 2
**Any kind of long-term care**	The highest income group	reference	reference
	The middle-income group	0.95 (0.78, 1.16)	1.00 (0.82, 1.23)
	The lowest income group	1.03 (0.83, 1.28)	1.13 (0.90, 1.41)
	p	0.702	0.464
**Specific type of long-term care**			
Institutional care only	The highest income group	reference	reference
	The middle-income group	0.73 (0.47, 1.14)	0.74 (0.47, 1.17)
	The lowest income group	0.80 (0.50, 1.28)	0.84 (0.51, 1.38)
	p	0.371	0.435
Home care only			
Estimate of use	The highest income group	reference	reference
	The middle-income group	0.96 (0.78, 1.19)	1.02 (0.82, 1.26)
	The lowest income group	1.01 (0.81, 1.27)	1.10 (0.87, 1.40)
	p	0.884	0.662
Monthly average hours	The highest income group		
	The middle-income group	0.67 (0.55, 0.82) *	0.69 (0.56, 0.85) *
	The lowest income group	0.98 (0.80, 1.21)	1.04 (0.83, 1.30)
	p	< 0.001	< 0.001

Any kind of long-term care and institutional care were analyzed with binary logistic regression and presented as odds ratio (95% confidence interval). Home care was analyzed with zero-inflated negative binomial regression. The estimate of use was presented as odds ratio (95% confidence interval). The monthly average hours of home care was presented as rate ratio (95% confidence interval).
Model 1: Adjusted for age, sex, living areas, living alone, income, Charlson Comorbidity Index, MMSE score.
Model 2: Additionally adjusted for education.
The lowest income group (n = 1749), annual income was between 64,848 SEK and 162,247 SEK. The middle-income group (n = 1745), annual income was between between 162,247 SEK and 209,036 SEK. The highest income group (n = 1746), annual income was more than 209,036 SEK.
p-value was calculated with Wald test to examine the overall significant association of education levels with outcomes.

REFERENCES

[1] Benchimol EI, Smeeth L, Guttmann A, Harron K, Moher D, Petersen I, Sørensen HT, von Elm E, Langan SM (2015) The REporting of studies Conducted using Observational Routinely-collected health Data (RECORD) statement. *PLoS Med* **12**, e1001885.

[2] World Health Organization (2015) *International statistical classification of diseases and related health problems*, World Health Organization, Geneva.

[3] World Health Organization Collaborating Centre for Drug Statistics Methodology, ATC/DDD Index 2021. https://www.whocc.no/atc_ddd_index/, Accessed on October 22, 2021.
